# Decitabine Increases the Transcription of *RIG-I* Gene to Suppress the Replication of Feline Calicivirus and Canine Influenza Virus

**DOI:** 10.3390/microorganisms13010143

**Published:** 2025-01-13

**Authors:** Shaotang Ye, Zhen Wang, Aolei Chen, Ying Chen, Gaoming Lou, Qingmei Xie, Gang Lu, Shoujun Li

**Affiliations:** 1 College of Veterinary Medicine, South China Agricultural University, Guangzhou 510642, China; yeshaotang@scau.edu.cn (S.Y.); zhenwang@outlook.com (Z.W.); chenolay@scau.edu.cn (A.C.); yingchen@outlook.com (Y.C.); 2 Henry Fok School of Biology and Agriculture, Shaoguan University, Shaoguan 512005, China; gaominglou@outlook.com; 3 Guangdong Provincial Key Laboratory of Utilization and Conservation of Food and Medicinal Resources in Northern Region, Shaoguan University, Shaoguan 512005, China; 4 College of Animal Science, South China Agricultural University, Guangzhou 510642, China; qmx@scau.edu.cn

**Keywords:** antiviral, decitabine, feline calicivirus, canine influenza virus, RIG-I

## Abstract

Developing novel antiviral drugs has always been a significant forefront in biological medicine research. Antiviral drugs can be extracted, purified, and synthesized from various biological sources and by different methods. However, they are less explored in veterinary medicine for animal viruses. This research primarily selected feline calicivirus (FCV) to screen the novel antiviral drug against animal viruses. A preliminary screening from a natural product library was conducted, with subsequent assessments to ascertain their toxicity levels and antiviral capabilities. The results showed that decitabine and alprostadil were effective in reducing FCV replication. The decitabine (5-aza-2′-deoxycytidine) was selected for antiviral mechanism investigation. Decitabine has been proven to modulate gene expression through its demethylating effect. Thus, we carried out further experiments and found that decitabine inhibited the FCV by enhancing the transcription of the feline *Retinoic acid-inducible gene I (RIG-I)* gene. Moreover, we also validated the same antiviral effect and mechanism of decitabine against the canine influenza virus (CIV). In summary, this study unveils the antiviral role of decitabine against FCV and CIV and provides evidence and novel insights into the demethylation drug-mediated antiviral effect for animal RNA viruses.

## 1. Introduction

Antiviral medications can be divided into two main categories based on their target specificity: those with specificity for specific viruses and those with broad-spectrum efficacy [1]. These compounds are derived from diverse sources, encompassing the realms of animal, plant, and microbial biology [2]. The scientific community has endeavored to identify various drugs that exhibit promising therapeutic potential against viral infections. Currently, these candidate drugs are in the preclinical evaluation phases, undergoing rigorous testing within cellular and animal models to ascertain their efficacy and safety [3]. However, these compounds are rarely explored in animal viral infection models.

Feline calicivirus (FCV), a prominent pathogen within the *Caliciviridae* family, is recognized for its ubiquity and capacity to incite respiratory afflictions in various feline species [4]. The evolution of this virus into a more pernicious form, known as virulent systemic disease (VSD), has been a subject of recent scientific scrutiny. VSD is characterized by its systemic nature and the severity of its symptoms, which can culminate in fatal outcomes for affected felines [5,6,7]. The current clinical paradigm for managing FCV infection is mainly palliative, focusing on alleviating symptoms through administering broad-spectrum antiviral agents. Several studies indicate that mefloquine, copper chloride, grape seed extract, and nitazoxanide have antiviral effects against non-VSD FCV [8,9,10,11]. However, the emergence of VSD has underscored the pressing need for clinical veterinary therapeutics. There is an exigent call for developing novel antiviral medications that are efficacious against both the conventional and virulent strains of FCV.

The canine influenza virus (CIV) belongs to the influenza A virus (IAV) of the *Orthomyxovirus* family [12]. Different subtypes of IAV can infect dogs and thus be considered the ″mixing vessel″ for the novel reassortant influenza A virus in the future to cause potential pandemics [10,11,12]. Hence, studies about antiviral drugs that modulate the canine innate immune response against CIV are important for developing antiviral strategies in the future. Nevertheless, the common preventative approach for CIV infection is vaccination, and there are no specific antiviral drugs for CIV infection.

Decitabine is a deoxycytidine analog that functions as a chemotherapeutic agent, primarily utilized for treating myelodysplastic syndromes. It operates by integrating into cellular DNA and impeding the activity of DNA methyltransferases, which decreases global DNA methylation and leads to changes in gene expression [13,14]. Recently, researchers discovered that decitabine shows varying levels of antiviral effects on the different viruses in vivo [15].

The development of antiviral drugs is a critical component of the “One Health” concept, which emphasizes reducing the impact of infective microorganisms on human health, animal health, and the economy. They are particularly crucial in the context of pandemics, such as the coronavirus and influenza outbreaks, where they can help lessen the severity of the disease, improve animal and human patient outcomes, and reduce the burden on healthcare systems [16,17].

In this study, we aim to match up with the “One Health” concept to develop more natural drugs for animals’ known or unknown RNA viruses. The feline calicivirus (FCV) and canine influenza virus (CIV) were used as models of animal RNA viruses for an antiviral effect investigation for calicivirus and influenza A virus. Our investigation aimed to identify novel antiviral compounds from a natural product library. After screening from a natural product library, the selected potential antiviral drug, decitabine, was evaluated to elucidate its toxicity, efficacy, and potential antiviral mechanism.

## 2. Materials and Methods

### 2.1. Cell, Virus, Natural Products, Antibodies, and Virus Titration

Crandell Rees Feline Kidney (CRFK) cells and Madin–Darby canine kidney (MDCK) cells were cultured at 37 °C and 5% (*v*/*v*) CO_2_ in Dulbecco’s Modified Eagle medium (DMEM; Biological Industries, Kibbutz Beit-Haemek, Israel) and 10% fetal bovine serum (Biological Industries). VSD-FCV-SCAU10 was propagated in CRFK cells [18]. CIV H3N2 (A/canine/Guangdong/02/2011) was propagated in specific pathogen-free chick embryos. The natural products library (L6000, Topscience, Shanghai, China) was stored at −20 °C; the details are listed in Supplementary S1. The antibodies used included Goat anti-Mouse IgG (H+L) Alexa Fluor 680 (AB175781, Abcam, Cambridge, UK), Goat anti-Rabbit IgG (H+L) Alexa Fluor 790 (AB175781, Abcam, Cambridge, UK), GAPDH antibody (GTX100118, GeneTex, Irvine, CA, USA), and rabbit polyclonal retinoic acid-inducible gene I (RIG-I) antibody (prepared by our laboratory). The virus was titrated as log10 (median tissue culture infectious dose [TCID_50_]/mL) and stored adequately at −80 °C. For FCV, the cytopathic effect (CPE) induced by FCV infection was used to calculate the virus titer. For CIV, the virus titer was calculated as previously mentioned [12].

### 2.2. Primary Screening of Antiviral Natural Products by FCV-Induced CPE Inhibition

CRFK cells were seeded in 96-well plates and grown in DMEM with 2% FBS. The confluent monolayer cells were infected with FCV-SCAU10 at a multiplicity of infection (MOI) of 0.01. After 1 h of virus inoculation, the cells were washed three times and replenished with new DMEM with 2% FBS containing different dilutions (10 μM, 50 μM, and 100 μM) of natural products. The CPE of the cells was observed under bright-light field microscopy and evaluated after 24 h of infection. The primary effective natural products would be tested in further investigations.

### 2.3. Evaluation of Cytotoxicity and Antiviral Efficacy of Natural Products

CRFK cells were seeded in 96-well plates and grown in DMEM with 2% FBS. To evaluate the cytotoxicity, the confluent monolayer cells were treated with new DMEM with 2% FBS containing different dilutions of natural products (ranging from 5 μM to 500 μM) for 24 h.

To evaluate antiviral efficacy against FCV, the confluent monolayer cells were infected with FCV-SCAU10 at a MOI of 0.01. After the virus inoculation, the cells were treated with new DMEM with 2% FBS containing different dilutions (ranging from 5 μM to 200 μM) of natural products for 24 h. When the treatment was finished, the cell viability was determined by CCK-8 (Beyotime, Shanghai, China) and calculated by the following equation: Cell viability = [OD (Compound) − OD (blank)]/[OD (control) − OD (blank)] × 100%. Then, the 50% cytotoxic concentration (CC_50_) and 50% inhibitory concentration (IC_50_) values were calculated using the inbuilt non-linear curve fitting functions following a log10 transformation of the natural product concentrations. Finally, the selectivity index (SI) was calculated as follows: SI = CC_50_/IC_50_.

To evaluate antiviral efficacy against CIV, the confluent monolayer MDCK cells were infected with CIV at a MOI of 0.1. The virus titer of the CIV was determined after the decitabine treatment.

The cytotoxicity and antiviral efficacy were evaluated by five replicates and three independent experiments.

### 2.4. RNA Extraction, cDNA Synthesis, and Quantitative Real-Time PCR (qRT-PCR)

The extracellular RNA of the cell supernatant was extracted using the PF Mag-Bind^®^ Viral DNA/RNA Kit (Omega Bio-Tek, Norcross, GA, USA). The intracellular RNA extraction, cDNA synthesis, and qRT-PCR tests were performed as previously described [12]. The copy number of FCV in extracellular was calculated according to the standard curve of the plasmid containing FCV ORF1. Primer sets used for the qRT-PCR were designed based on the published sequences and are listed in Supplementary S2.

### 2.5. RNA Interference

Small interfering RNA (siRNA) targeting the feline RIG-I and canine RIG-I were synthesized by RiboBio (Guangzhou, China). CRFK or MDCK cells were transfected with 100 nmol of the indicated siRNA. The relative expression of feline and canine RIG-I, FCV, and CIV were evaluated by qRT-PCR, respectively.

### 2.6. Western Blotting

The cells were lysed using Cell lysis buffer for Western blotting and IP (P0037, Beyotime). Lysates were collected and centrifuged at 15,000× *g* rpm/min for 10–15 min. A total of 30 μg of each sample was separated by SDS-PAGE and transferred to a PVDF membrane blocked for at least 10 min with QuickBlock Blocking Buffer for Western blotting (P0252, Beyotime) at room temperature for 15 min and then incubated overnight at 4 °C with primary antibodies. After washing with PBST, secondary antibodies were incubated for 1 h at room temperature. Protein bands were visualized by Odyssey Sa (Li-cor, Lincoln, NE, USA).

### 2.7. Bioinformatic Analysis of the CpG Island of Canine and Feline RIG-I Gene

To predict the CpG island of the canine and feline *RIG-I* genes, the full-length mRNA of the canine *RIG-I* gene (XM_005626701.4, CDS region on nucleotides 169 to 2946) and feline *RIG-I* gene (XM_006939199.5, CDS region on nucleotides 187 to 2976) were analyzed by MethPrime (https://www.urogene.org/methprimer/) (accessed on 24 July 2024).

### 2.8. Statistical Analysis

All data were analyzed by the unpaired Student’s *t*-test using Prism v10.3 software (GraphPad, San Diego, CA, USA), indicating the mean ± SD; * *p <* 0.05, ** p < 0.01, and *** p < 0.001.

## 3. Results

### 3.1. Primary Screening of Antiviral Natural Products Against Feline Calicivirus

**Figure 1:**
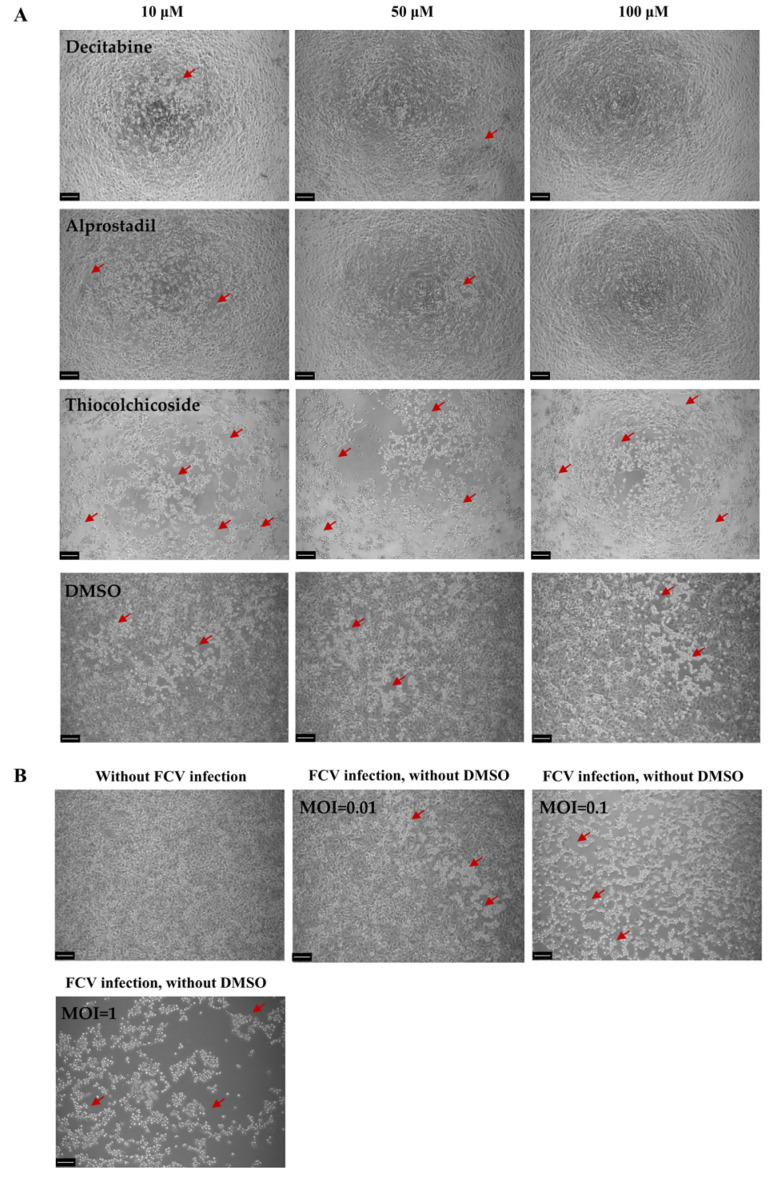
Primary screening of antiviral natural products against feline calicivirus. (**A**) Indicates the bright-field observation of FCV-induced CPE (MOI = 0.01) in CRFK cells, which were treated with different concentrations of natural products, including decitabine, alprostadil, tubercidin, thiocolchicoside, and DMSO, respectively. DMSO was used as the non-antiviral effect control with FCV infection. Scale bar, 100 μM. (**B**) Indicates the mock group, including no FCV infection and FCV infection without DMSO treatment. Scale bar, 100 μM. Red arrow indicates the CPE sites.

Feline calicivirus (FCV) remains a prevalent respiratory pathogen in cats. While studies have shown that copper chloride, mefloquine, and nitazoxanide can significantly suppress FCV replication, no specific drugs are currently available for clinical FCV infection [8,9,10]. To address this issue and discover potential candidate drugs for clinical treatment, we conducted a rapid screening of a natural product library (160 drugs). The library contained natural products from various sources, such as animals, plants, organisms, microorganisms, and others. These natural products were diluted into 10, 50, and 100 μM and were co-incubated for 24 h after FCV inoculation. Through the primary evaluation of the antiviral effect of CPE inhibition, we identified six potentially effective natural products: decitabine, alprostadil, luteolin, tubercidin, trans-3-inndoleacrylicacid, and mangiferin (Figure 1 and Figure S1). Further research on these natural products may lead to the development of effective drugs for FCV treatment.

### 3.2. Evaluation of Cytotoxicity and Antiviral Efficacy of Natural Products

**Figure 2:**
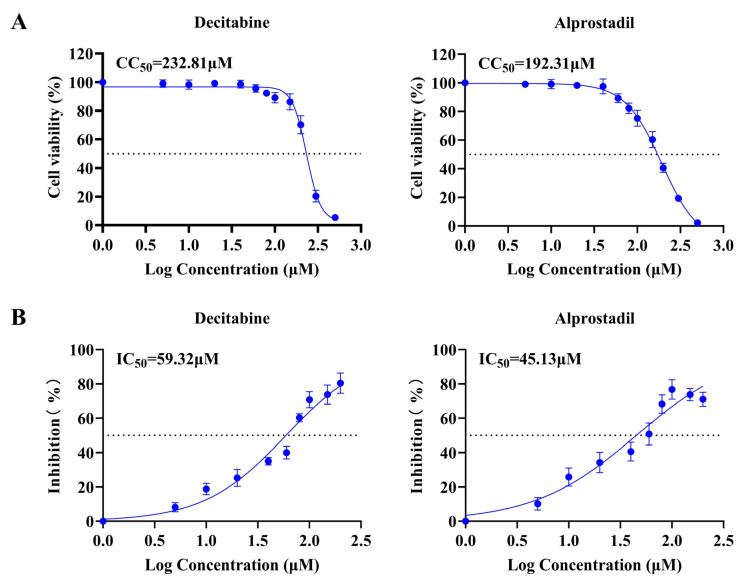
Evaluation of cytotoxicity and antiviral efficacy of natural products in vitro. (**A**) The CC_50_ values of decitabine and alprostadil were conducted. (**B**) The IC_50_ values of decitabine and alprostadil were evaluated.

**Figure 3:**
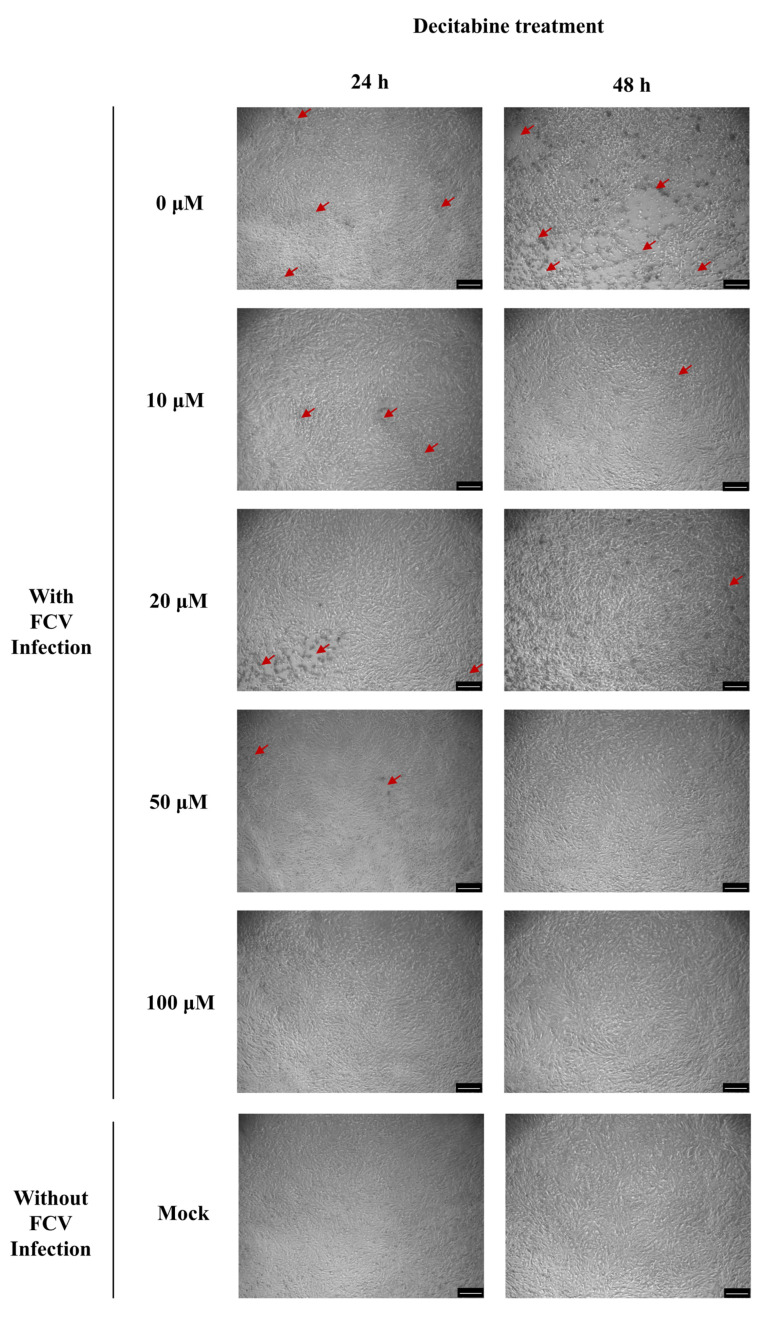
The FCV-induced CPE observation to confirm the antiviral effect with different times and concentrations of decitabine treatment. Red arrow indicates the CPE sites. Scale bar, 250 μM.

In the preceding section, we identified six natural products that exhibit potential effects against FCV infection. To further investigate their antiviral properties, we conducted assays in vitro to evaluate their cytotoxicity and antiviral efficacy. Our findings indicate that decitabine and alprostadil have CC_50_ values of 232.81 μM and 192.31 μM on CRFK cells, respectively (Figure 2A). This suggested that decitabine and alprostadil are less toxic to feline cells and more suitable for further antiviral research. Subsequently, we conducted antiviral efficacy assays to determine the IC_50_ values of 59.32 μM and 45.13 μM for decitabine and alprostadil, respectively (Figure 2B). We also carried out a CPE observation to confirm the antiviral effect with different times and concentrations of decitabine treatment. The results showed that decitabine reduced FCV-induced CPE in a time- and concentration-dependent manner (Figure 3). Based on the formula, the SI values for decitabine and alprostadil were 3.92 and 4.26, respectively. In conclusion, our results indicate that decitabine and alprostadil exhibit promising potential as antiviral drugs for FCV infection.

### 3.3. Potential Role of Decitabine Against Feline Calicivirus

A recent study has revealed that the application of decitabine in neuroblastoma cells resulted in the activation of an innate immune response mediated by the retinoic acid-inducible gene I (RIG-I) [19]. This protein, capable of detecting viral RNA, initiates the RIG-I-like receptors pathway, leading to the production of type I interferon to combat viral infections. Numerous studies have demonstrated the antiviral efficacy of RIG-I against calicivirus [20,21]. According to previous research, the innate immune system of felines plays a crucial role in combating various viral infections [22,23]. This led us to focus on the relationship among the FCV, feline RIG-I, and decitabine.

**Figure 4:**
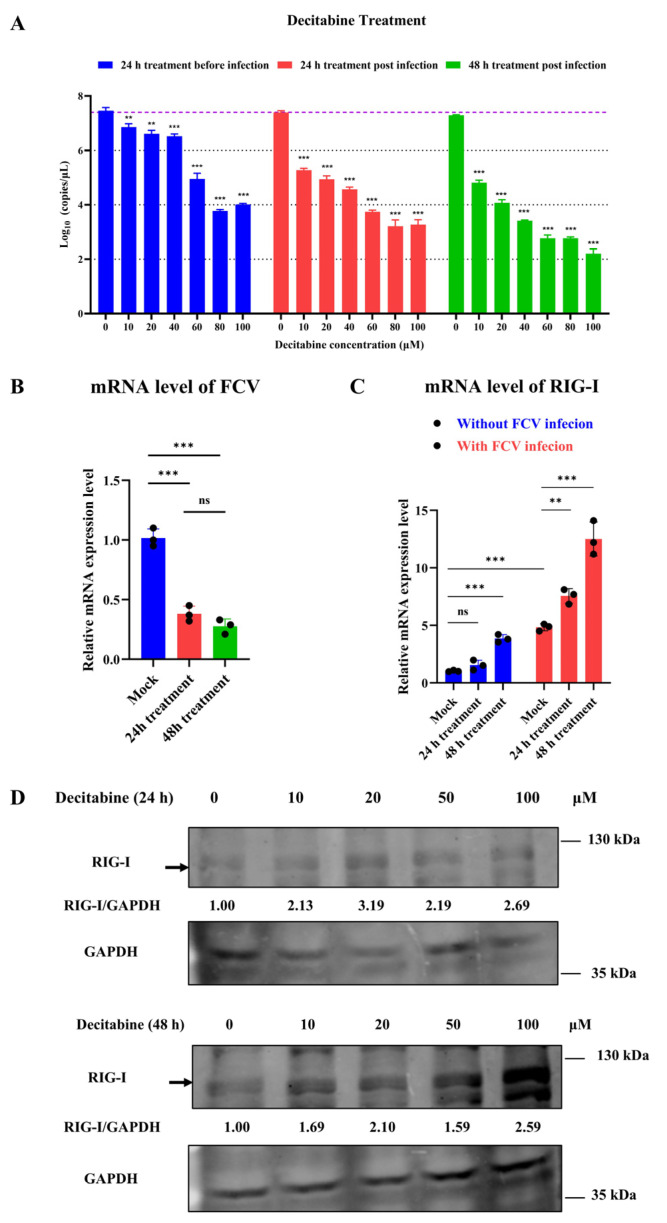
Investigation of the potential antiviral role of decitabine against feline calicivirus. (**A**) The extracellular FCV RNA of CRFK cells under decitabine treatment was evaluated. (**B**) The intracellular FCV RNA of CRFK cells treated with decitabine was extracted, and the relative level was determined. (**C**) The relative level of the feline *RIG-I* gene in CRFK cells under decitabine treatment with and without FCV infection was determined. Samples were analyzed by qRT-PCR in three independent experiments. (ns indicated no significance, ** *p* < 0.01, *** *p* < 0.001) (**D**) The protein expression of feline RIG-I in CRFK cells treated with different concentrations of decitabine was determined.

To further validate the antiviral efficacy of decitabine against FCV, we conducted a qRT-PCR to determine the copy number of the FCV in extracellular and relative expression of intracellular RNA under different concentrations of decitabine treatment. The results demonstrated that decitabine exhibited the highest antiviral effect against FCV replication in the group treated for 48 h post-infection, which is consistent with our previous CPE observation assays (Figure 4A,B).

**Figure 5:**
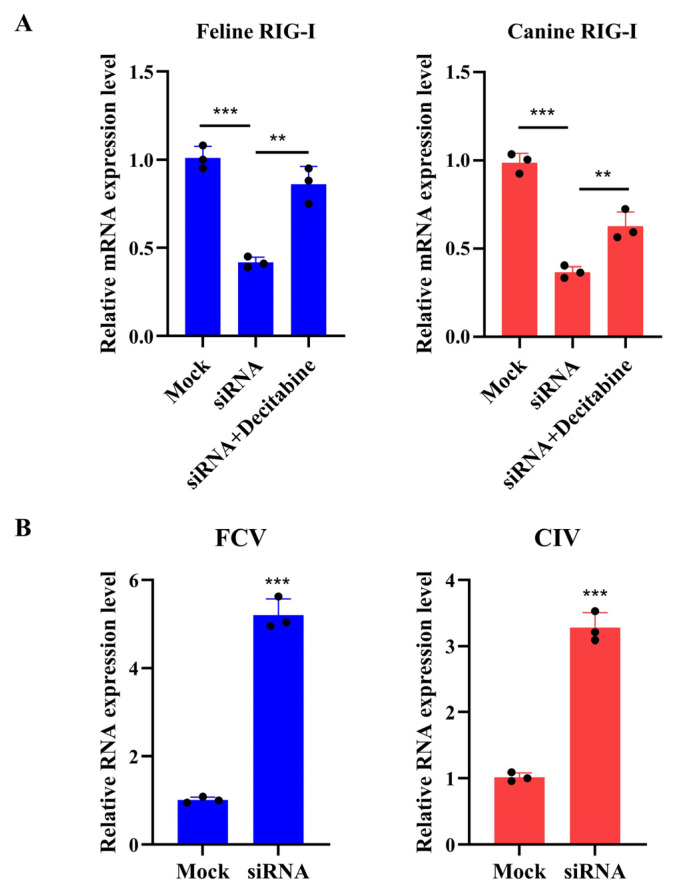
Further validation of the antiviral effect of RIG-I against feline calicivirus and canine influenza virus. (**A**) Cells were transfected with siRNA for feline and canine *RIG-I* gene for 24 h, and one of the RNA interference groups was treated with 100 μM decitabine after transfection. The relative level of the *RIG-I* gene in cells was detected. (**B**) Cells were transfected with siRNA for feline and canine *RIG-I* gene for 24 h, and cells were infected with FCV and CIV for 24 h post 24 h transfection, respectively. The relative levels of FCV and CIV were detected, respectively. (**A**,**B**) Samples were analyzed by qRT-PCR in three independent experiments. (** *p* < 0.01, *** *p* < 0.001).

To investigate the potential mechanism of decitabine against FCV, we treated CRFK cells (with or without FCV infection) with 50 μM decitabine. We measured the mRNA level of the feline *RIG-I* gene using a qRT-PCR after 24 or 48 h. The results showed that 48 h of treatment with decitabine without FCV infection led to an increased mRNA level of the feline *RIG-I* gene. Notably, FCV infection could trigger the activation of feline RIG-I transcription, and the use of decitabine at two different concentrations significantly increased the expression of *RIG-I* in the CRFK cells infected with FCV (Figure 4C). We then conducted Western blotting to confirm the reliability of the decitabine-mediated protein expression of feline RIG-I. Our findings demonstrated that 24 h of treatment with decitabine at higher concentrations slightly increased protein expression. In comparison, 48 h of treatment at all concentrations significantly stimulated the protein expression of the feline *RIG-I* gene (Figure 4D). These results suggested that decitabine could mediate the expression of feline RIG-I in a dose- and time-dependent manner. In addition, we carried out an experiment where the siRNA for feline RIG-I was transfected into CRFK cells. We found that decitabine could partially recover the mRNA level of feline RIG-I against gene silencing (Figure 5A). We also validated that the silenced expression of feline RIG-I could significantly promote the replication of FCV (Figure 5B). In summary, the decitabine-mediated activation of the feline *RIG-I* transcription might represent a potential strategy for preventing FCV infection.

### 3.4. Decitabine Enhances the Transcription of Canine RIG-I to Inhibit the Replication of Canine Influenza Virus

**Figure 6:**
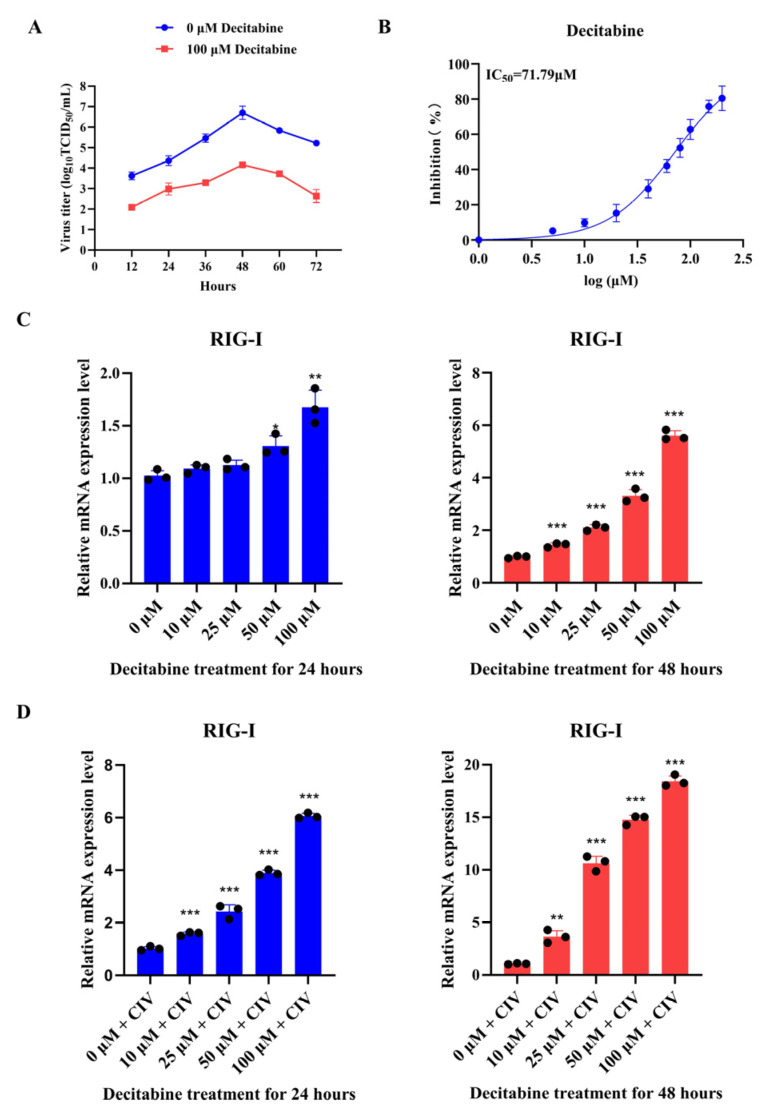
Investigation of potential antiviral role of decitabine against canine influenza virus. (**A**) CIV growth kinetics were evaluated in MDCK cells treated with 100 μM decitabine. (**B**) The IC_50_ of decitabine against CIV was determined. (**C**) The relative level of the canine *RIG-I* gene in MDCK cells was treated at different times and concentrations of decitabine without CIV infection. (**D**) The relative level of the canine *RIG-I* gene in MDCK cells was treated at different times and concentrations of decitabine with CIV infection. (**C**,**D**) Samples were analyzed by qRT-PCR in three independent experiments. (* *p* < 0.05, ** *p* < 0.01, *** *p* < 0.001).

The previous experiments related to FCV had proven the antiviral effect of decitabine in feline cells. To investigate whether decitabine could perform the same antiviral effect by modulating RIG-I expression in canine cells, we carried out a series of experiments with canine influenza virus (CIV). We first determined the virus titer with decitabine treatment, and the results showed that decitabine could significantly inhibit CIV replication (Figure 6A). Then, we measured the IC_50_ value of decitabine against CIV, which was determined as 71.79 μM (Figure 6B). These results suggested that decitabine could also perform antiviral effects against CIV. Our previous study has indicated that canine RIG-I inhibits CIV, and we proved it in this study (Figure 5B) [12]. This evidence prompted us to investigate decitabine’s antiviral role against CIV. Different times and concentrations of decitabine were conducted on MDCK cells with or without CIV infection. The results showed that decitabine could significantly increase canine *RIG-I* transcription under a 24 h treatment of 100 μM and 48 h with any concentration (Figure 6C). We also found that decitabine could upregulate the canine *RIG-I* transcription under 24 and 48 h treatments with any concentration in the CIV infection assay (Figure 6D). We verified that decitabine could partially recover the mRNA level of canine RIG-I after the transfection of siRNA (Figure 5A). These results proved that decitabine could regulate the canine *RIG-I* transcription to inhibit CIV replication.

### 3.5. Bioinformatic Analysis of the Potential Methylation on Canine and Feline RIG-I Gene

**Figure 7:**
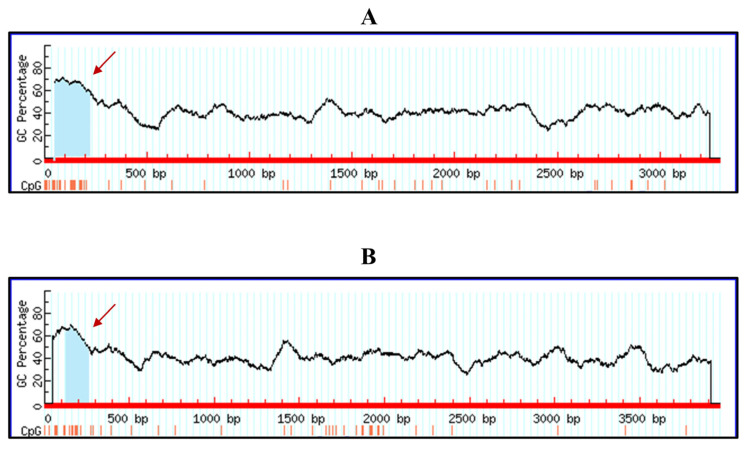
Bioinformatic analysis of the CpG island of canine and feline *RIG-I* gene. (**A**) indicated the analysis for canine *RIG-I* gene. (**B**) indicated the analysis for feline *RIG-I* gene. The red arrow indicates the CpG island.

Our previous experiments indicated that decitabine could regulate canine and feline *RIG-I* transcription. Decitabine is well known for the demethylation of DNA to regulate gene expression. To further investigate the regulation mechanism of decitabine for the RIG-I gene, we analyzed the potential methylation on the canine and feline *RIG-I* genes. Bioinformatic analysis suggested a CpG island in the non-CDS region of these two full-length gene transcription mRNAs, respectively (Figure 7). These CpG islands on the canine and feline *RIG-I* genes might be responsible for the decitabine-induced antiviral effect against CIV and FCV, respectively.

## 4. Discussion

Since the early 20th century, the identification of antiviral compounds has expanded the clinical approaches against viral diseases. One of the earliest antiviral drugs, idoxuridine, was approved in 1963, marking the beginning of a new era in antiviral therapy [24]. For instance, acyclovir, an acyclic guanosine analog, is widely used to treat herpesvirus infections by inhibiting viral replication [25]. A derivative, valacyclovir, offers improved bioavailability but must be used cautiously due to potential adverse effects such as nephrotoxicity [26]. Since then, the focus has been on identifying specific targets to increase selectivity and reduce the side effects. Natural products and their derivatives have been invaluable in this pursuit. Consequently, exploring natural products can potentially lead to finding more antiviral drugs for treating viral infections in animals upon testing, which may pave the way for more effective and targeted treatments in veterinary medicine.

FCV belongs to the *Caliciviridae* family and has similar properties to human norovirus and rabbit haemorrhagic disease virus [10,21,27,28,29]. Previous studies have indicated that different subtypes of the avian influenza virus could infect dogs [30,31,32], and these suggest that dogs may serve as the “mixing vessel” for generating novel mixed recombinations. Pursuing the “One Health”, FCV and CIV act as two suitable models in this study. Therefore, we first carried out a natural product library screening, and we found that decitabine and alprostadil both showed low toxicity to feline cells and a significant inhibitory effect on FCV. We subsequently chose decitabine, a deoxycytidine analog, for further investigation in this study. The antiviral mechanism of alprostadil in feline cells will be further investigated in the future.

The antiviral properties of decitabine are attributed to its ability to modulate gene methylation levels and gene expression status, as evidenced by its effectiveness against human immunodeficiency virus, human hepatitis viruses, and equid herpesvirus-1 [15,33]. Moreover, it has been found to induce dsRNA transcription from endogenous retroviruses, thereby eliciting an innate immune response to inhibit feline leukemia virus [34]. Although decitabine exhibits a broad range of antiviral activity, the precise mechanism by which it exerts its effects against specific viruses remains unclear.

In this study, we found that decitabine could increase the transcription of feline and canine *RIG-I* genes in a time- and concentration-dependent manner and this enhancement could be more potent during infection. We also found that decitabine was available to partially recover the transcription of feline and canine RIG-I from gene silencing. These phenomena are similar to previous studies., which indicate that decitabine acts as a modulating agent for the immune response of mammalian animals [19,34]. Combined with the bioinformatic analysis of the full-length mRNA of feline and canine RIG-I, the CpG island was shown in the non-CDS region. This predictive result may be helpful for subsequent validation investigations through methylation-specific PCR or bisulfite sequencing PCR in the future.

However, we still could not conclude that decitabine can inhibit RNA viruses (calicivirus and influenza A virus) by only regulating the transcription of the *RIG-I* gene. It might even be expanded to the innate immune response or other host-pathogen interactions, such as the interferon system. Decitabine has also been identified to induce the RIG-I-like pathway by modulating mitochondrial stress in neuroblastoma [19]. These results suggest that decitabine can enhance the monitoring effect of RIG-I to sense cancer cell origin-dsRNA and RNA virus-induced dsRNA. Recent studies have demonstrated that decitabine is able to trigger the innate antiviral response against equid herpesvirus 4 and inhibit herpes simplex virus type 1 by inducing lethal mutagenesis [35,36]. Furthermore, viral infections are increasingly recognized as potent modulators of the host’s epigenetic landscape [37,38,39,40]. Thus, future studies could focus on the antiviral mechanism of decitabine to delve deeper into whether decitabine has more gene regulatory functions on the host’s epigenetic landscape for innate immune responses.

## 5. Conclusions

In summary, this study carried out a preliminary and expeditious screening of 160 natural products to evaluate their antiviral efficacy against FCV. The findings demonstrated that decitabine and alprostadil exhibited a substantial antiviral effect against FCV while exhibiting minimal cytotoxicity to CRFK cells in vitro. Our investigation led to the proposal of a hypothesis: decitabine modulates the expression of the cytoplasmic viral sensor, feline RIG-I, to inhibit FCV replication. This study also provided evidence of the decitabine-induced upregulation of canine RIG-I expression to suppress CIV infection. This study offered novel insights into the development of natural antiviral products against companion animal viruses and uncovered the potential antiviral role of decitabine against FCV and CIV in vitro.

## Data Availability

The original contributions presented in the study are included in the article/Supplementary Materials, further inquiries can be directed to the corresponding authors.

## Supplementary Materials

The following supporting information can be downloaded at: https://www.mdpi.com/article/10.3390/microorganisms13010143/s1, Supplementary S1: All natural products used in this study are listed in detail. Supplementary S2: The qPCR primers and siRNA sequences used in this study are listed in detail, as well as the primary screenings of other antiviral natural products against feline calicivirus.

## References

[1] Martinez J.P., Sasse F., Brönstrup M., Diez J., Meyerhans A. (2015). Antiviral Drug Discovery: Broad-Spectrum Drugs from Nature. Nat. Prod. Rep..

[2] Vougogiannopoulou K., Corona A., Tramontano E., Alexis M.N., Skaltsounis A.-L. (2021). Natural and Nature-Derived Products Targeting Human Coronaviruses. Molecules.

[3] Li G., Hilgenfeld R., Whitley R., De Clercq E. (2023). Therapeutic Strategies for COVID-19: Progress and Lessons Learned. Nat. Rev. Drug Discov..

[4] Pesavento P.A., Chang K.-O., Parker J.S.L. (2008). Molecular Virology of Feline Calicivirus. Vet. Clin. N. Am. Small Anim. Pract..

[5] Hofmann-Lehmann R., Hosie M.J., Hartmann K., Egberink H., Truyen U., Tasker S., Belák S., Boucraut-Baralon C., Frymus T., Lloret A. (2022). Calicivirus Infection in Cats. Viruses.

[6] Guo H., Miao Q., Zhu J., Yang Z., Liu G. (2018). Isolation and Molecular Characterization of a Virulent Systemic Feline Calicivirus Isolated in China. Infect. Genet. Evol..

[7] Pesavento P.A., Maclachlan N.J., Dillard-Telm L., Grant C.K., Hurley K.F. (2004). Pathologic, Immunohistochemical, and Electron Microscopic Findings in Naturally Occurring Virulent Systemic Feline Calicivirus Infection in Cats. Vet. Pathol..

[8] McDonagh P., Sheehy P.A., Fawcett A., Norris J.M. (2015). Antiviral Effect of Mefloquine on Feline Calicivirus in Vitro. Vet. Microbiol..

[9] Li D., Cui Z., Li G., Zhang L., Zhang Y., Zhao H., Zhang S., Guo Y., Zhao Y., Men F. (2020). Antiviral Effect of Copper Chloride on Feline Calicivirus and Synergy with Ribavirin in Vitro. BMC Vet. Res..

[10] Joshi S.S., Su X., D’Souza D.H. (2015). Antiviral Effects of Grape Seed Extract against Feline Calicivirus, Murine Norovirus, and Hepatitis A Virus in Model Food Systems and under Gastric Conditions. Food Microbiol..

[11] Cui Z., Li D., Xie Y., Wang K., Zhang Y., Li G., Zhang Q., Chen X., Teng Y., Zhao S. (2020). Nitazoxanide Protects Cats from Feline Calicivirus Infection and Acts Synergistically with Mizoribine In Vitro. Antivir. Res..

[12] Wang Z., Ye S., Yao C., Wang J., Mao J., Xu L., Liu Y., Fu C., Lu G., Li S. (2021). Antiviral Activity of Canine RIG-I against Canine Influenza Virus and Interactions between Canine RIG-I and CIV. Viruses.

[13] Greve G., Schüler J., Grüning B.A., Berberich B., Stomper J., Zimmer D., Gutenkunst L., Bönisch U., Meier R., Blagitko-Dorfs N. (2021). Decitabine Induces Gene Derepression on Monosomic Chromosomes: In Vitro and In Vivo Effects in Adverse-Risk Cytogenetics AML. Cancer Res..

[14] Li L., Liu W., Sun Q., Zhu H., Hong M., Qian S. (2021). Decitabine Downregulates TIGAR to Induce Apoptosis and Autophagy in Myeloid Leukemia Cells. Oxidative Med. Cell. Longev..

[15] Xiao J., Liu P., Wang Y., Zhu Y., Zeng Q., Hu X., Ren Z., Wang Y. (2022). A Novel Cognition of Decitabine: Insights into Immunomodulation and Antiviral Effects. Molecules.

[16] Jamrozik E., Selgelid M.J. (2020). COVID-19 Human Challenge Studies: Ethical Issues. Lancet Infect. Dis..

[17] Li Y.-T., Linster M., Mendenhall I.H., Su Y.C.F., Smith G.J.D. (2019). Avian Influenza Viruses in Humans: Lessons from Past Outbreaks. Br. Med. Bull..

[18] Mao J., Ye S., Li Q., Bai Y., Wu J., Xu L., Wang Z., Wang J., Zhou P., Li S. (2022). Molecular Characterization and Phylogenetic Analysis of Feline Calicivirus Isolated in Guangdong Province, China from 2018 to 2022. Viruses.

[19] Lin H.-Y., Chuang J.-H., Wang P.-W., Lin T.-K., Wu M.-T., Hsu W.-M., Chuang H.-C. (2020). 5-Aza-2′-Deoxycytidine Induces a RIG-I-Related Innate Immune Response by Modulating Mitochondria Stress in Neuroblastoma. Cells.

[20] Mao J., Ye S., Deng J., Song J., Wang Z., Chen A., Zhou P., Li S. (2023). Feline Calicivirus P39 Inhibits Innate Immune Responses by Autophagic Degradation of Retinoic Acid Inducible Gene I. Int. J. Mol. Sci..

[21] Dang W., Xu L., Yin Y., Chen S., Wang W., Hakim M.S., Chang K.-O., Peppelenbosch M.P., Pan Q. (2018). IRF-1, RIG-I and MDA5 Display Potent Antiviral Activities against Norovirus Coordinately Induced by Different Types of Interferons. Antivir. Res..

[22] Tian J., Kang H., Huang J., Li Z., Pan Y., Li Y., Chen S., Zhang J., Yin H., Qu L. (2020). Feline Calicivirus Strain 2280 P30 Antagonizes Type I Interferon-Mediated Antiviral Innate Immunity through Directly Degrading IFNAR1 mRNA. PLoS Pathog.

[23] Yumiketa Y., Narita T., Inoue Y., Sato G., Kamitani W., Oka T., Katayama K., Sakaguchi T., Tohya Y. (2016). Nonstructural Protein P39 of Feline Calicivirus Suppresses Host Innate Immune Response by Preventing IRF-3 Activation. Vet. Microbiol..

[24] De Clercq E., Li G. (2016). Approved Antiviral Drugs over the Past 50 Years. Clin. Microbiol. Rev..

[25] O’Brien J.J., Campoli-Richards D.M. (1989). Acyclovir: An Updated Review of Its Antiviral Activity, Pharmacokinetic Properties and Therapeutic Efficacy. Drugs.

[26] D’Antonio F., Marinceu D., Prasad S., Khalil A. (2023). Effectiveness and Safety of Prenatal Valacyclovir for Congenital Cytomegalovirus Infection: Systematic Review and Meta-analysis. Ultrasound Obstet. Gynecol..

[27] Guo H., Zhu J., Miao Q., Qi R., Tang A., Liu C., Yang H., Yuan L., Liu G. (2020). RPS5 Interacts with the Rabbit Hemorrhagic Disease Virus 3′ Extremities Region and Plays a Role in Virus Replication. Vet. Microbiol..

[28] Buckley D., Dharmasena M., Fraser A., Pettigrew C., Anderson J., Jiang X. (2018). Efficacy of Silver Dihydrogen Citrate and Steam Vapor against a Human Norovirus Surrogate, Feline Calicivirus, in Suspension, on Glass, and on Carpet. Appl. Environ. Microbiol..

[29] Zhu J., Miao Q., Tang J., Wang X., Dong D., Liu T., Qi R., Yang Z., Liu G. (2018). Nucleolin Mediates the Internalization of Rabbit Hemorrhagic Disease Virus through Clathrin-Dependent Endocytosis. PLoS Pathog..

[30] Wang G., Dos Anjos Borges L.G., Stadlbauer D., Ramos I., Bermúdez González M.C., He J., Ding Y., Wei Z., Ouyang K., Huang W. (2019). Characterization of Swine-Origin H1N1 Canine Influenza Viruses. Emerg. Microbes Infect..

[31] Sun H., Blackmon S., Yang G., Waters K., Li T., Tangwangvivat R., Xu Y., Shyu D., Wen F., Cooley J. (2017). Zoonotic Risk, Pathogenesis, and Transmission of Avian-Origin H3N2 Canine Influenza Virus. J. Virol..

[32] Falchieri M., Reid S.M., Dastderji A., Cracknell J., Warren C.J., Mollett B.C., Peers-Dent J., Schlachter A.-L.D., Mcginn N., Hepple R. (2024). Rapid Mortality in Captive Bush Dogs (*Speothos venaticus*) Caused by Influenza A of Avian Origin (H5N1) at a Wildlife Collection in the United Kingdom. Emerg. Microbes Infect..

[33] Bouchard J., Walker M.C., Leclerc J.M., Lapointe N., Beaulieu R., Thibodeau L. (1990). 5-Azacytidine and 5-Azadeoxycytidine Inhibit Human Immunodeficiency Virus Type 1 Replication in Vitro. Antimicrob. Agents Chemother..

[34] Greggs W.M., Clouser C.L., Patterson S.E., Mansky L.M. (2012). Discovery of Drugs That Possess Activity against Feline Leukemia Virus. J. Gen. Virol..

[35] Bautista L., Sirimanotham C., Espinoza J., Cheng D., Tay S., Drayman N. (2024). A Drug Repurposing Screen Identifies Decitabine as an HSV-1 Antiviral. Microbiol. Spectr..

[36] Normand C., Thieulent C.J., Fortier C., Sutton G., Senamaud-Beaufort C., Jourdren L., Blugeon C., Vidalain P.-O., Pronost S., Hue E.S. (2024). A Screening Study Identified Decitabine as an Inhibitor of Equid Herpesvirus 4 That Enhances the Innate Antiviral Response. Viruses.

[37] Zhang Y., Zhang L.-S., Dai Q., Chen P., Lu M., Kairis E.L., Murugaiah V., Xu J., Shukla R.K., Liang X. (2022). 5-Methylcytosine (m^5^C) RNA Modification Controls the Innate Immune Response to Virus Infection by Regulating Type I Interferons. Proc. Natl. Acad. Sci. USA.

[38] Sun H., Tu S., Luo D., Dai C., Jin M., Chen H., Zou J., Zhou H. (2023). Protein Arginine Methyltransferase 5 Mediates Arginine Symmetric Dimethylation of Influenza A Virus PB2 and Supports Viral Replication. J. Med. Virol..

[39] Narayan P., Ayers D.F., Rottman F.M., Maroney P.A., Nilsen T.W. (1987). Unequal Distribution of N6-Methyladenosine in Influenza Virus mRNAs. Mol. Cell. Biol..

[40] Courtney D.G., Kennedy E.M., Dumm R.E., Bogerd H.P., Tsai K., Heaton N.S., Cullen B.R. (2017). Epitranscriptomic Enhancement of Influenza A Virus Gene Expression and Replication. Cell Host Microbe.

