# Supplementary material for: Decitabine Increases the Transcription of RIG-I Gene to Suppress the Replication of Feline Calicivirus and Canine Influenza Virus
Source: Microorganisms. 2025 Jan 13;13(1):143. doi: 10.3390/microorganisms13010143 (PMC11767338; doi:10.3390/microorganisms13010143)
Supplement: Supplementary file 1 [file microorganisms-13-00143-s001.zip › Supplementary 2.pdf]

Table S1 Primer pairs used for quantitative RT-PCR analysis.

Primer	Sequence of oligonucleotides (5′ → 3)	Gene accession NO.
Feline RIG-I-qF	CACTGTTGAAGCGTTTACAACC	XM_006939199.5
Feline RIG-I-qR	ACCTGCATAATTTCTTCACATTCCT	
Feline GAPDH-qF	ACGGGAAACTTGTCATCAATGGAAAGC	NM_001009307.1
Feline GAPDH-qR	AGACTCCACAACATACTCAGCACCAG	
Canine RIG-I-qF	GTTGCTGATGAAGGCATCGACATTG	XM_005626701
Canine RIG-I-qR	CACTTGCTACCTCTTGCTCTTCCTC	
Canine GAPDH-qF	AATTCCACGGCACAGTCAAGGC	NM_001003142
Canine GAPDH-qR	ACAACATACTCAGCACCAGCATCAC	
FCV-qF	TAACTCGGTTTTTGATTTGGCCTGGGCT	
FCV-qR	CATATGCGGCTCTGATGGCTTGAAACTG	
CIV-qF	TGATCCTCTCGTTATTGCCGCAAG	
CIV-qR	CACTCTGCTGTTCCTGCCGATAC	

Table S2 siRNA used for gene knock-down.

Primer	Sequence of oligonucleotides (5′ → 3)
Feline RIG-I-	TGAGTAAACTACACCCCAA
Canine RIG-I	TGAGTAAGTTACACCCTAA

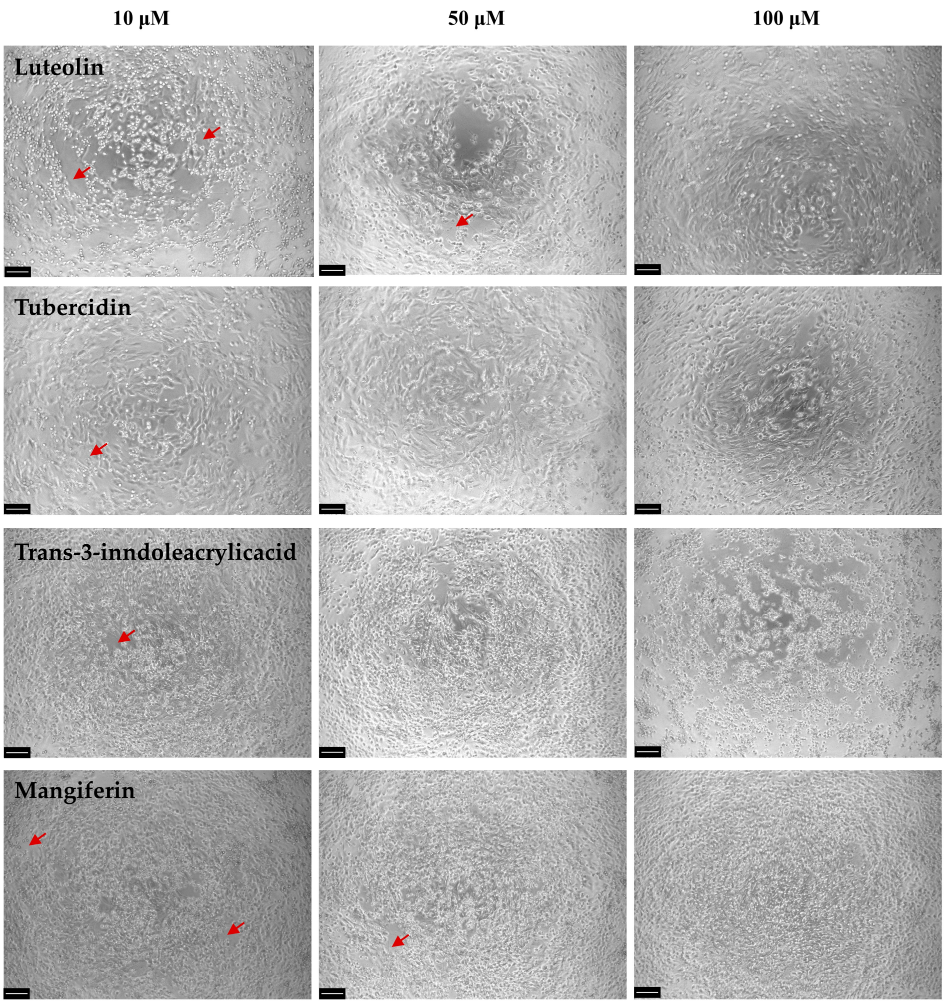

Figure S1. Primary screening of antiviral natural products against feline calicivirus, including luteolin, tubercidin, trans-3-inndoleacrylicacid and mangiferin. Scale bar, 100 μm. Red arrow indicated the CPE sites.
